# History of a Case of Hydrophobia, Not Arising from an Ascertained Cause

**Published:** 1831

**Authors:** Daniel Drake

**Affiliations:** Cincinnati


Art. III.—*History of a Case of Hydrophobia, not arising from an ascertained cause.*—By Daniel Drake, M. D.

The symptoms, termination, and morbid appearance, on dissection, of the following case, prove it to have been true hydrophobia; (*rabies;*) and, still, the unfortunate young man had not been bitten by an animal of any kind. The case seems, therefore, worthy of being reported.

The patient, Josiah Morehead, jr., by trade a tanner, was 23 years of age. On Sunday, the first of May, 1831. complained of feeling unwell, without indicating to his friends any particular symptoms, except a general muscular soreness, and oppressed breathing. On Monday morning, said he had not rested well the night before, and did not rise as usual. At seven o'clock, his mother carried him a cup of coffee. On taking the saucer in his hand, and carrying it towards his lips, he jerked his head back; and after making two or three unsuccessful efforts, concluded that the steam made him start. The coffee was then cooled, and offered to him again, but with the same result; and the repetition produced some slight spasms of the muscles of the upper part of the body. Neither he nor his friends, at this time, seem, however, to have thought of hydrophobia; but Dr. Woolley was immediately called in. Finding his pulse active, and his tongue furred, the doctor bled him to 16 or 20 ounces, and ordered him 30 grains of calomel, incorporated with powdered sugar. He was calm and rational; but it was not without much difficulty, that he succeeded in licking up and swallowing, in the most gradual manner, the dry powder. His spasmodic agitation increasing, Professor Cobb, Dr. Robert Moorhead, and myself, were sent for, and first saw him about the middle of the day. The experiment of offering him drinks was repeated, in our presence; but he could not suffer them to approach his mouth; and the attempt excited most painful and universal agitation, attended with sudden screams, and great, but undefinable alarm. His pulse was rapid. No delirium existed, and he made constant efforts to keep himself composed. The question of hydrophobia having been started in the family, he was, for the purpose of tranquilizing his feelings, assured that he did not labor under that disease. An enema was ordered, which agitated him very much, and with difficulty he succeeded in swallowing a dose of calomel and opium, in powder. In the afternoon, he lost about 30 ounces of blood. At bed time, a considerable degree of emphysema was discovered near his clavicles, and on the sides of his neck. Through the night, he took a quantity of sulphate of morphia with sugar.

On Tuesday morning, we found that he had slept none. The calomel had operated once or twice, copiously. His agitation and sudden startings were somewhat mitigated, but the water-dread was unabated; and after repeated trials, having the fluid both in open and covered vessels, we gave up all further attempts. In these experiments, his own exertions were great and persevering. He would carry the vessel, slowly and obliquely, towards his mouth; but as it approached his lips, his head would suddenly recede, his countenance assume an ex-expression of alarm and horror, and the muscles of his chest, neck, and arms, generally, take on a spasmodic action, producing an exceedingly embarrassed respiration. His breathing was, indeed, constantly straitened, and, occasionally, he coughed; which, taken in connexion with the external emphysema, suggested the possibility that a lesion of the bronchia had taken place; which, permitting the extravasation of air in the neighborhood of the œsophagus and trachea, might, *possibly*, occasion dysphagia and dyspnœa. Under this impression, I attempted, in the afternoon, to examine him with the stethoscope; but, notwithstanding his utmost efforts to lie still, the touch of the cylinder excited so much flurry, that no successful observation could be made. No further administration of medicine was now attempted, but cupping beneath the clavicles was ordered. Very little blood was drawn, and the operation greatly increased his spasmodic agitation. Before night, he started incessantly, in the utmost terror; the slightest touch, even of his hair, would excite a universal muscular contraction—greatest, however, in the muscles of respiration—and it became necessary to surround his bed with several resolute persons, to keep him in it. He frothed at the mouth, but manifested no disposition to bite, and retained his senses so perfectly, as to be conscious of his approaching end, and make rational, Christian preparation to meet it. Two or three hours before his death, he shook hands with his friends, and bade them farewell; not long after which, his agitations began to subside, and it soon became obvious that he was *in articulo mortis*. He expired about 8 o'clock in the evening, 36 hours after the dread of water first manifested itself.

Examination of the body 19 hours after death, by Prof. Cobb, in the presence of several medical gentlemen.—Corpse stiff. Complexion, a dark sallow. Extensive emphysema of the subcutaneous parts—greatest, about the throat and neck, but extending to the shoulders and the whole of the chest. On raising the sternum, which led to no escape of air, the cellular tissue of the anterior medrastinum was found in the same condition, as was, also, the cellular substance around the trachea and œsophagus. Considerable portions of the pericardium, and of the pleura covering the diaphragm, were dry. The lungs collapsed, and were not emphysematous, except a small portion near the heart. The mucous membrane of the larynx, trachea, and bronchia, was highly inflamed, and covered with frothy mucus. No orifice could be discovered, through which the air had escaped. The heart was sound. The lining membrane of the pharynx, like that of the trachea, was inflamed; that of the œsophagus healthy; that of the stomach nearly natural, but the organ contained a pint of dark colored fluid, though he had drunk nothing for 48 hours. Most of this was, *perhaps*, the secretion from the trachea and bronchia.—

5

The liver, spleen and pancreas were healthy. No other parts of the body were examined.

The phenomena and morbid anatomy of this, as of most cases of hydrophobia, show that the parts which are supplied by the nerves of respiration, were the chief seats of the disease.—Thus, the muscles affected with spasms, were chiefly the respiratory; the emphysema was confined to the chest and neck; a great deal of dyspnœa, from spasmodic action, was present, throughout the whole course of the disease; and, on dissection, the mucous membrane of the pharynx, larynx, trachea, and bronchia, was found to have been inflamed. The late Professor Dorsey has reported, in the Medical Museum, Vol. 4, a case, which proved fatal under circumstances, that suggested to those who saw it, the opinion, that the patient died from closure of the rima glottidis by spasmodic action.

The learned authors of the Dict. de Sci. Med., art. *Rage*, hold the following language in reference to the seat of this malady:

> "The scalpel carried into the aerial passages—the larynx, trachea, and bronchia, discloses traces of inflammation, which are the more manifest, the further we descend into the organ. In the bronchial ramifications, the mucous membrane has the color of the lees of wine. In four dissections, we found frothy mucus in the bronchia, and either in the larynx or trachea. This mucus was mixed with a little blood in the bronchia of one of these subjects, while it was as white as snow in another."

Other parts, according to these gentlemen, are generally free from the traces of disease.

The aerial passages, then, are the seats of the frothy discharge from the mouths of rabid animals; and this froth constitutes the virus that propagates the disease. If it should happen not to be present in the mouth of the animal at the moment when he bites, the malady will not be communicated.

If we regard the disease with which Morehead died, as a case of true hydrophobia, how can we explain its origin? Did it arise independently of the infection of a rabid animal? Has such a case as this, ever thus arisen, in the human subject, or in any animal, not of the natural orders, which embrace the cat and dog? This question, it would seem, must be answered in the negative. In reference to the present case, the only explanation which has suggested itself to my own mind, or to that of my colleagues, is the following.—For the last year, canine madness has been, in some degree, endemic in and around Cincinnati. At least one person, has died in an adjoining county, from the bite of a mad dog; and many domestic animals have been lost from the same cause. The skin of one of these, having the hair besmeared with the frothy discharge from the animal's mouth, had probably been taken to the tannery, where this young man was an operative. Six or eight weeks before his death, he was burnt on one of his fingers, and at the time of the attack, the sore was still covered with a scab. To this sore the infection, thus conveyed, might have been applied, and occasioned the disease.

But to this theory of its origin, may be opposed the prevailing belief of the profession, that the morbid secretions of herbivorous animals, cannot communicate the disease. Is this, however, the fact? Is not the testimony on this subject chiefly negative, and therefore, inconclusive? I am not aware, that animals which do not bite when angry, are prone to do so, when affected with hydrophobia; and hence, the possibility of their communicating the disease may not have been demonstrated. The secretions of their mucous membranes, inflamed in hydrophobia, may be poisonous; but are not likely often to be inoculated, simply, because such animals are not prone to bite. If the discharge from the mouth of a man affected with true hydrophobia, can excite the disease, I see no conclusive reason, why that from an animal purely herbivorous, may not. The communication of the vaccine disease from the cow to the human subject, and the mortality of dogs and cats, during the prevalence of certain epidemics among our species, indicate conclusively, the existence of several identities between the human race and certain genera of the lower animals. It has been questioned, however, whether the bite of a rabid man *can* communicate the disease. But from the dread which this malady inspires in all who are called to the bed-side of such patients, as well as from the care and solicitude of the infected, it is not probable that they have often attempted, or been allowed, to bite others. Moreover, the following experiment, by two distinguished physiologists, would seem to establish the affirmative of the proposition:

"On the 19th of June, 1813, in the Hotel Dieu, of Paris, Messrs. Magendie and Brechet, impregnated a bit of linen rag, with the saliva of a man who died of hydrophobia, a few minutes afterwards; and, carrying it about twenty paces from the bed-side of the patient, inoculated two dogs with it. One of them went mad on the 27th of July, and bit two others, of which one became rabid on the 26th of August."—*Dict. des Sciences Medicales, Art. Rage.*

From this experiment, conducted in presence of a class of medical students, I feel justified in concluding, that the discharge from the mouth of a rabid man, may be infectious, and, by analogy, am led to believe, that herbivorous quadrupeds may, likewise, propagate the disease. If that of the unfortunate patient, Morehead, was not thus excited, I can offer no explanation of its origin.

A few weeks after its occurrence, I heard of a case, which, from report, seemed likely to throw some light on this obscure subject. A fisherman, named Clark, was said to have been bitten by a rabid horse, and actually to labor under hydrophobia. He was in the care of several of the "Steam, or Botanical Doctors," who have, for the last few years, been epidemic in the northern and western states. Obtaining permission to visit the patient, I saw him three times, in company with other medical gentlemen; and satisfied myself, as they also were satisfied, that his disease was not hydrophobia. Several months before, in attempting to drench his horse, which was said to have been bitten by a mad dog, he received a slight wound, which gave him great alarm, and excited in his system, a decided state of nervous irritation. All his sensibilities became morbid, and distressing apprehensions of the result, were awakened and sustained. A species of hysteria, in short, was the consequence; and in this condition, he at last took to his bed, and summonned the "steamers," who, for two or three weeks, surrounded him with hot bricks, and drenched him with a decoction of lobelia inflata, which, with various other medicated draughts, he, drank without difficulty. The case, if it proved any thing, went to support the opinion, that an herbivorous animal cannot communicate the disease.

*Cincinnati, September*, 1831.

